# Immune Thrombocytopenia: A Rare Adverse Event of Vancomycin Therapy

**DOI:** 10.7759/cureus.39348

**Published:** 2023-05-22

**Authors:** Emeka S Obi, Devdat LNU, Norense O Ehimwenma, Opeyemi Tobalesi, Winifred Iklaki, Faiza Arslan

**Affiliations:** 1 Department of Health Administration, College of Public Health, East Tennessee State University, Johnson City, USA; 2 Department of Gastroenterology, Dartford and Gravesham NHS Trust, Dartford, GBR; 3 Medical Education, Liaquat University of Medical and Health Sciences, Jamshoro, PAK; 4 Department of Internal Medicine, Diana, Princess of Wales Hospital, Grimsby, GBR; 5 Department of Internal Medicine, College of Health Sciences, University of Ilorin, Ilorin, NGA; 6 Department of Internal Medicine, All Saints University School of Medicine, Roseau, DMA; 7 Department of Internal Medicine, Rawalpindi Medical University, Rawalpindi, PAK

**Keywords:** diabetic foot infections, immune-mediated thrombocytopenia, drug-induced immune thrombocytopenia, vancomycin-induced immune thrombocytopenia, vancomycin

## Abstract

Vancomycin, a glycopeptide antibiotic, is widely used for Gram-positive cocci or bacilli bacteria-induced serious infections. Although considered safe and effective, it still causes adverse events. Vancomycin-induced immune thrombocytopenia is a rarely reported adverse event, manifesting from asymptomatic thrombocytopenia to life-threatening bleeding. We underline a case of a 56-year-old male with a diabetic foot with an infected exudating purulent ulcer. He experienced a significant drop in platelet count after commencing vancomycin, and discontinuing vancomycin resulted in improved platelet count with positive vancomycin-induced anti-platelet antibodies. After ruling out other possible causes of thrombocytopenia, a presumptive diagnosis of vancomycin-induced thrombocytopenia was made.

## Introduction

Vancomycin, a glycopeptide antibiotic, is mainly used to treat Gram-positive cocci or bacilli bacteria-induced serious infections and bacterial infections diseases resistant to antibiotics, especially methicillin-resistant Staphylococcus aureus (MRSA) and coagulase-negative Staphylococcus [1]. Although considered safe and effective, it still causes adverse events. Ototoxicity and nephrotoxicity are well-known side effects. Other adverse reactions of vancomycin therapy include eosinophilia, erythrocyte syndrome, systemic symptoms, and allergic reactions [2]. Vancomycin-associated immune thrombocytopenia is a rare adverse reaction and is not widely reported in the literature [3,4]. We report a case of immune thrombocytopenia induced by vancomycin therapy after ruling out all other possible etiologies, including autoimmune and viral screening. Due to the frequency of vancomycin use in hospital settings, our case highlights the importance of early recognition and management of rare, vancomycin-induced immune thrombocytopenia.

## Case presentation

**Figure 1:**
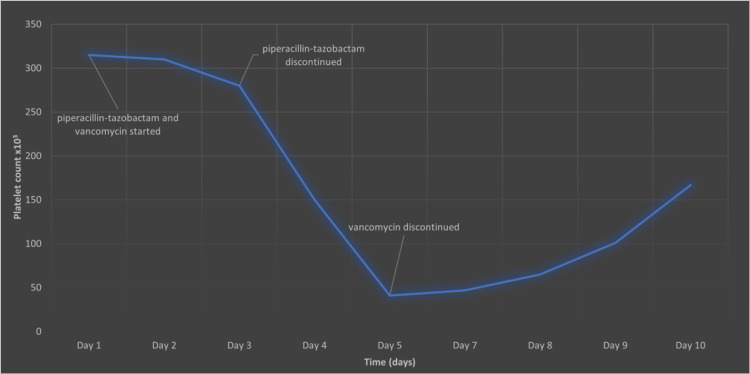
Platelet count during hospital stay in relation to vancomycin use.

A 56-year-old male was admitted to our hospital for evaluation and management of his left diabetic foot for the last three weeks. He also complained of high-grade fever, fatigue, and nausea. He was a known case of diabetes mellitus and hypertension and underwent total hip replacement on the right side three years ago. He was not compliant with his medications, including amlodipine and sitagliptin. On examination, there was an infected purulent ulcer with exudates and surrounding erythema on the plantar surface of the left big toe with the second and third toe involvement. Pulses were palpable in both feet. He was febrile (100 F) and tachycardic (101/minute) with a respiratory rate of 20/minute and blood pressure of 100/70 mmHg. Respiratory, neurological, musculoskeletal, and cardiovascular examinations were unremarkable. His initial laboratory results showed leukocytosis (14,000/mm3), mild anemia (hemoglobin: 10.2 g/dl), elevated erythrocyte sedimentation rate (26/hr), and C-reactive protein (7.2 mg/dl) with normal platelet count. He underwent debridement and drainage of the abscess, and infected tissue was noted to involve the phalanx and metatarsal bone. He was empirically commenced on IV piperacillin-tazobactam of 13.5 g/day and vancomycin of 20 mg/kg every 12 hours, and infected tissue was sent for culture. On day 3, blood and wound culture was positive for MRSA, and the patient was switched to vancomycin along with supportive management, including IV fluid and antipyretics. On admission day 5, his clinical condition improved; however, his platelet count dropped to 41,000/mm3 (Figure 1).

A repeat investigation on day 6 revealed a further drop in platelet count. He did not report any bleeding episode from any site except for indicative of petechiae, including pinpoint, non-blanching, purpuric lesions measuring approximately 1-3 mm in diameter on the lower extremity. Peripheral smear revealed very few platelets with normal morphology and no schistocytes. Bleeding time, prothrombin time, activated prothrombin time, serum creatinine, and total bilirubin were within the normal range (Table 1).

**Table 1:** Results of laboratory evaluations on admission day 6.

Parameter	Lab value	Reference range
White cell count	13500 /mm^3^	(4000-11000)/mm^3^
Hemoglobin	10 g/dl	(14-18) g/dl
Bilirubin	1.1 mg/dl	(0.1-1.2) mg/dl
Bleeding time	6 minutes	(1-9) minutes
Platelet count	41,000/mm^3^	(150,000-450,000)/mm^3^
Prothrombin time	12 seconds	(10-13) seconds
Activated prothrombin time	29 seconds	(21-35) seconds
Serum creatinine	0.9 mg/dl	(0.7-1.3) mg/dl
Serum calcium	9.9 mg/dl	(9.0-10.6) mg/dl
Blood urea nitrogen	14 mg/dl	(08-24) mg/dl
Alanine aminotransferase	49 IU/L	(8-57) IU/L
Alkaline phosphatase	77 mg/dl	(36-95) mg/dl
C-reactive protein	6 mg/dl	(0.3-1) mg/dl
Erythrocyte sedimentation rate	25/hour	(0-20)/hour
Lactate dehydrogenase	167 IU/L	(140-280) IU/L

Vancomycin was immediately stopped, and he was commenced on IV meropenem of 500 mg/12 hour and daptomycin of 6 mg/kg/day. A provisional diagnosis of drug-induced immune thrombocytopenia was made as there was no thrombocytopenia on patient presentation. Thrombocytopenia developed after initiation of vancomycin, and further investigation to rule out other etiologies were performed, including acute viral hepatitis, HIV, and autoimmune screening, which were negative. Flow cytometry was positive for vancomycin-induced platelet antibodies. Platelet count started improving over the following days on subsequent evaluations, and the patient completed his course of daptomycin and meropenem with no relapse of thrombocytopenia (Figure 1).

## Discussion

Immune thrombocytopenia may present clinically from asymptomatic thrombocytopenia to life-threatening bleeding and can cause substantial morbidity and mortality in critically ill patients [5]. There are several factors and mechanisms which can induce immune thrombocytopenia. Severe infections, autoimmune diseases, bone marrow suppression, and medications are among the common causes [6]. Drug-induced immune thrombocytopenia is a fatal clinical syndrome with a high risk of bleeding caused by drug-dependent platelet antibodies causing accelerated platelet destruction [7]. Commonly reported drugs causing immune thrombocytopenia include diuretics, quinines, anticonvulsants, sulfonamides, disease-modifying antirheumatic drugs (DMARDs), and nonsteroidal anti-inflammatory drugs (NSAIDs) [8]. Although rare, vancomycin is also implicated in the etiology of drug-induced immune thrombocytopenia. We have tabulated the reported cases of vancomycin-induced immune thrombocytopenia after searching the PubMed database (Table 2) [3,4,7,9-11].

**Table 2:** Reported cases of vancomycin-induced immune thrombocytopenia. NR: Not reported; M: Male; F: Female.

Author et al.	Age/sex	Indication	Duration of use (days)	Platelet nadir (10^3^/ml)	Time to nadir (days)	Time to recovery (days)	Platelet antibodies
MacDougall KN et al. [3]	81/M	Infected prosthesis	10	1	3	6	Positive
Hameed M et al. [4]	74/M	Sepsis	11	70	6	NR	NR
Ruggero MA et al. [7]	63/M	Diabetic foot	14	2	15	7	Positive
Anand A et al. [9]	54/M	Cellulitis	6	100	6	2	Positive
Lobo N et al. [10]	67/M	Pneumonia	3	2	4	2	Positive
Candemir B et al. [11]	54/F	Implantable hematoma	15	49	12	4	NR

The pathophysiology of vancomycin-induced immune thrombocytopenia is complex, involving both immune-mediated and direct toxic effects on the platelets. In an immune-mediated mechanism, the body’s own immune response mistakenly targets and destroys the platelets in response to the presence of vancomycin [12]. This process occurs after the binding of vancomycin to platelet membrane glycoprotein, which forms a hapten-carrier complex. This complex triggers the production of antibodies against the platelet-vancomycin complex, leading to the destruction of platelets by the reticuloendothelial system. Consequently, this increases the risk of bleeding, which can manifest as ecchymosis, epistaxis, or more severe bleeding events [8]. Vancomycin may also have a direct toxic effect on platelets. High concentrations of vancomycin in the serum may cause platelet aggregation and activation, leading to the release of pro-coagulant and inflammatory factors, including p-selectin and platelet factor 4 [10]. These factors may contribute to a prothrombotic state, increasing the risk of thrombosis and further aggravating the decrease in platelet count. Other contributing factors may include prolonged treatment duration, the presence of underlying hematological disorders, or autoimmune diseases, which may make patients more susceptible to developing immune thrombocytopenia in response to vancomycin [3]. Diagnosis of vancomycin can be delayed as the clinical presentation can be mistaken for other causes of immune thrombocytopenia. Laboratory findings may include a sudden onset drop in platelet count, drug-dependent platelet antibodies against immune complex on flow cytometry, and a rise in platelet count after discontinuing vancomycin [13]. Management of vancomycin-induced thrombocytopenia involves the immediate discontinuation of the drug, which often leads to a rapid improvement in platelet count. However, in severe cases with life-threatening bleeding, additional interventions may be required, which include platelet transfusion, corticosteroids, or IV immunoglobulins. An alternative therapy should be considered to treat the underlying infection to avoid further complications [14]. Our patient presented with an infected diabetic foot managed with piperacillin-tazobactam and vancomycin. He was found to have severe asymptomatic thrombocytopenia (platelet count: <51,000/mm3), which improved after discontinuing vancomycin therapy. The presence of drug-dependent platelet antibodies on flow cytometry provides a temporal relationship of thrombocytopenia following vancomycin exposure in the absence of other causes.

## Conclusions

Vancomycin-induced immune thrombocytopenia is a rare adverse reaction and commonly the evidence of misdiagnosis. Because of high morbidity and mortality, this condition requires immediate evaluation with serial platelet count and platelet antibodies. Immediate discontinuation of vancomycin, including transfusion, steroids, and immunoglobulin, should be started immediately based on the patient's clinical status. In confirmed cases, the patients are advised to avoid vancomycin in the future. As vancomycin is used frequently in clinical practice today, physicians should be aware of this potentially life-threatening condition.
